# A parenting programme to prevent abuse of adolescents in South Africa: study protocol for a randomised controlled trial

**DOI:** 10.1186/s13063-016-1452-8

**Published:** 2016-07-19

**Authors:** Lucie Cluver, Franziska Meinck, Yulia Shenderovich, Catherine L. Ward, Rocio Herrero Romero, Alice Redfern, Carl Lombard, Jenny Doubt, Janina Steinert, Ricardo Catanho, Camille Wittesaele, Sachin De Stone, Nasteha Salah, Phelisa Mpimpilashe, Jamie Lachman, Heidi Loening, Frances Gardner, Daphnee Blanc, Mzuvekile Nocuza, Meryn Lechowicz

**Affiliations:** Centre for Evidence-Based Intervention, Department of Social Policy & Intervention, University of Oxford, Barnett House, 32 Wellington Square, Oxford, OX1 2ER UK; Department of Psychiatry and Mental Health, University of Cape Town, Cape Town, South Africa; Institute of Criminology, University of Cambridge, ᅟ, ᅟ; Department of Psychology, University of Cape Town, Cape Town, South Africa; Biostatistics Unit, South African Medical Research Council, Cape Town, South Africa; Clowns Without Borders South Africa, PO Box 18670, Durban, 4014 South Africa; UNICEF, Offices of Research – Innocenti, Florence, Italy

**Keywords:** Child abuse, Parenting, Low- and middle-income countries

## Abstract

**Background:**

An estimated one billion children experience child abuse each year, with the highest rates in low- and middle-income countries. The Sinovuyo Teen programme is part of Parenting for Lifelong Health, a WHO/UNICEF initiative to develop and test violence-prevention programmes for implementation in low-resource contexts. The objectives of this parenting support programme are to prevent the abuse of adolescents, improve parenting and reduce adolescent behavioural problems. This trial aims to evaluate the effectiveness of Sinovuyo Teen compared to an attention-control group of a water hygiene programme.

**Methods/Design:**

This is a pragmatic cluster randomised controlled trial, with stratified randomisation of 37 settlements (rural and peri-urban) with 40 study clusters in the Eastern Cape of South Africa. Settlements receive either a 14-session parenting support programme or a 1-day water hygiene programme. The primary outcomes are child abuse and parenting practices, and secondary outcomes include adolescent behavioural problems, mental health and social support. Concurrent process evaluation and qualitative research are conducted. Outcomes are reported by both primary caregivers and adolescents. Brief follow-up measures are collected immediately after the intervention, and full follow-up measures collected at 3–8 months post-intervention. A 15–24-month follow-up is planned, but this will depend on the financial and practical feasibility given delays related to high levels of ongoing civil and political violence in the research sites.

**Discussion:**

This is the first known trial of a parenting programme to prevent abuse of adolescents in a low- or middle-income country. The study will also examine potential mediating pathways and moderating factors.

**Trial registration:**

Pan-African Clinical Trials Registry PACTR201507001119966. Registered on 27 April 2015. It can be found by searching for the key word ‘Sinovuyo’ on their website or via the following link: http://www.pactr.org/ATMWeb/appmanager/atm/atmregistry?_nfpb=true&_windowLabel=BasicSearchUpdateController_1&BasicSearchUpdateController_1_actionOverride=%2Fpageflows%2Ftrial%2FbasicSearchUpdate%2FviewTrail&BasicSearchUpdateController_1id=1119

**Electronic supplementary material:**

The online version of this article (doi:10.1186/s13063-016-1452-8) contains supplementary material, which is available to authorized users.

## Background

Worldwide, an estimated one billion children experience abuse each year [1], with the highest rates in low- and middle-income countries (LMICs) and in particular the World Health Organization (WHO) Africa region [2]. Although prevalence data are limited, new studies suggest increases in abuse during adolescence [3], which is also a time of important social, emotional and continued neural development [4].

**Fig. 1:**
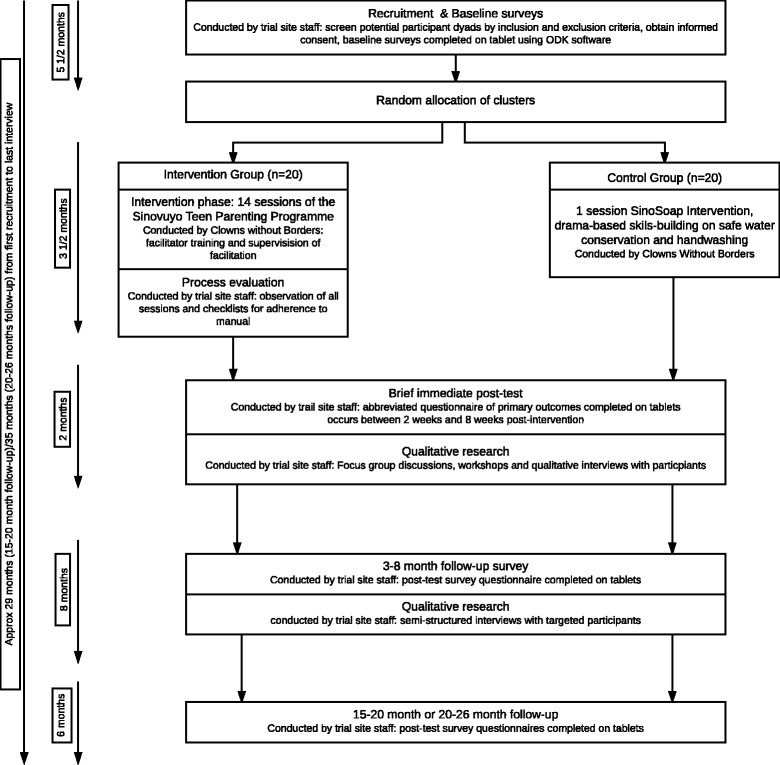
Trial flow chart

Despite this, systematic reviews find that the vast majority of research into child abuse prevention is in high-income countries and with younger children [5]. In LMICs, three trials have tested abuse prevention parenting programmes, two targeted at children under 10 years old in South Africa [6] and Liberia [7] and one for 13-year-olds and under in Burundi [8]. To date, there are no known randomised trials of a parenting programme to prevent abuse of adolescents in any LMIC [9].

Existing evidence – albeit from different contexts and child age groups – demonstrates good effect sizes of group-based parenting programmes that are grounded in social learning theory, problem-solving and acquisition of behaviour management skills [10]. Indeed, a recent systematic review showed high transportability of parenting programmes to address problem behaviour amongst younger children across high-income countries and contexts [11].

However, three major limitations exist to transportability to an LMIC. Firstly, many existing evidence-based programmes charge fees for training and manuals, making costs prohibitive for low-resource agencies and governments [12]. Other programmes require qualified health professionals for implementation, who are not available in the highest-need areas. Finally, many have technological components (e.g. videos and web-based modalities) that are as yet inaccessible in areas with poor electricity and internet access.

In response, an international collaboration was started in 2012, to develop and rigorously test a suite of child abuse prevention programmes for different age groups. Parenting for Lifelong Health (PLH) includes WHO, the United Nations Children’s Emergency Fund (UNICEF) and academics from the global South and North, with donor partners, LMIC governments and the President’s Emergency Plan for AIDS Relief (PEPFAR)-US Agency for International Development (USAID). PLH programmes are developed with participatory input from families in LMICs, for implementation by lay community workers, and have minimal equipment requirements. If shown effective in randomised trials, programmes will be freely available under licensing that prohibits any commercial or profit interests.

The adolescent programme, called Sinovuyo (‘we have joy’) Teen, has undergone incubation development and testing in very low-income rural and peri-urban areas of South Africa. Initial development used systematic reviews to identify effective components [10], input provided voluntarily from over 50 international academics and practitioners, and in-depth qualitative work with adolescents and caregivers [6]. Draft programme manuals were written in partnership between a local non-governmental organisation (NGO; Clowns Without Borders South Africa) and academics from the universities of Oxford and Cape Town.

A first pilot pre-post non-controlled test with 30 caregiver–adolescent dyads showed initial reductions in abuse and adolescent behavioural problems, and no evidence of harm [13]. Concurrent qualitative research identified participant requests to incorporate economic strengthening approaches into the programme, to lessen family conflict over money. After adaptation, a second pre-post non-controlled test with 115 dyads showed reductions in abuse, behavioural problems, depression and caregiver (but not adolescent) substance abuse, and improvements in positive/involved parenting and social support [14]. This second stage also piloted family budgeting and savings sessions, which were subsequently incorporated into the third version of the manual. The studies also found unanticipated high levels of programme dissemination within communities, particularly through church groups and community meetings.

At all stages, the project has been a close partnership between NGOs, the South African Department of Social Development and UNICEF. These agencies intend to decide whether to implement the programme within a provincial and national rollout, based on the results of this trial. Furthermore, an additional 17 countries in Sub-Saharan Africa, Central Asia, Eastern Europe and the Middle East have expressed strong interest in implementing the programme. The timing of this trial is thus critical for policy-making for child abuse prevention in LMICs.

Due to extenuating circumstances (high levels of civil and political unrest in the research sites in the first 6 months of the trial), this protocol is submitted after recruitment of the participants, and, therefore, falls outside the journal’s usual policies. Data collection is still underway at the time of submission.

## Methods/design

In this pragmatic cluster randomised trial in real-world settings, 40 rural and peri-urban settlements, containing 600 caregiver–adolescent dyads, are randomised to two parallel arms.

### Study Aims

The study aims to compare the effectiveness of a 14-session caregiver and adolescent programme with an attention-control group, amongst high-risk adolescents aged 10–18 and their families. Primary outcomes for the trial are (1) harsh and abusive discipline and (2) parenting. Secondary outcomes include adolescent externalising behaviour, parenting stress, mental health and social support. Exploratory outcomes include family financial coping, avoiding risk in the community, sexual harassment/abuse and educational engagement.

### Inclusion criteria

#### Communities

The communities are rural and peri-urban settlements within a 1-hour driving distance of King William’s Town, in the Eastern Cape province of South Africa. All areas have high rates of unemployment, poor infrastructure and high HIV/AIDS prevalence [15].

#### Participants

Adolescents are between 10 and 18, and either sleep in the same dwelling for at least four nights a week as their primary caregiver or have regular contact with them. Adolescents and their primary caregivers gave informed consent to participate. Recruitment followed pragmatic trial principles of closely approximating methods of inclusion in NGO or government services. Families were referred by a range of social services, schools and local chieftains, and were also able to self-refer as struggling with an adolescent. All families completed a brief screening questionnaire asking if there were regular arguments in the home.

### Exclusion criteria

Following pragmatic trial principles, there were no exclusion criteria for families. There were no requirements for a biological relationship between caregiver and adolescent. Communities required approval from local traditional or political leaders (chieftains and ward councillors), and were estimated to be safe enough (during daylight hours and with local support) to hold a parenting group without serious risk to the participants. If a participant had such severe learning disabilities that they were unable to consent to participation, they were not included in the study for ethical reasons.

### Control sites

Control rural and peri-urban settlements receive a one-session hygiene programme called SinoSoap. This is implemented by the NGO Clowns Without Borders South Africa and involves drama-based skills-building on safe water conservation and handwashing for children. All participating families in control sites receive a ‘hope soap’: a bar of soap containing a small toy that is only accessible when the soap is used.

### Intervention sites

Intervention rural and peri-urban settlements receive the 14-session parenting programme called Sinovuyo Teen. Weekly sessions take place in the communities (church and community halls, schools and under trees).

### Programme and training

The intervention programme is implemented by locally recruited unqualified community members and local social auxiliary workers. All programme facilitators received 1 week’s initial training and weekly supervisions from Clowns Without Borders South Africa. The training was participatory and activity-based, and training materials are being developed for free availability.

The programme is based on evidence-informed parenting principles, such as praising each other, managing anger and stress, joint problem-solving, non-violent discipline, rules and routines, keeping adolescents safe in the community, and responding to crises (see Table 1). It uses collaborative problem-solving techniques (not didactic methods) and traditional stories, role-play, modelling and stress reduction activities [16–18].

**Table 1:** Overview of intervention session topics

Session	Content	Mode
1	Introducing the programme and defining participant goals	Joint
2	Building a positive relationship through spending time together	Joint
3	Praising each other	Joint
4	Talking about emotions	Separate
5	What do we do when we’re angry?	Separate
6	Problem-solving: putting out the fire	Joint
7	Motivation to save and making a budget with our money	Joint
8	Dealing with problems without conflict I	Separate
9	Dealing with problems without conflict II	Separate
10	Establishing rules and routines	Joint
11	Ways to save money and making a family saving plan	Joint
12	Keeping safe in the community	Joint
13	Responding to crisis	Joint
14	Widening circles of support	Joint

Ten programme sessions are joint with caregivers and adolescents, and four sessions have separate components to allow sensitive discussions. Participants are encouraged to engage in home practice in the week following each session. For participants unable to attend sessions due to illness, disability etc., *khaya* (home) catch-ups are arranged to give brief session content at home or in the hospital. A simple lunch is included at the beginning of each session as many participants find concentration difficult due to hunger.

### Randomisation

Stratified randomisation was used. There were 40 eligible study clusters, 32 rural and eight peri-urban clusters, representing the two strata. Complete randomisation was done for clusters within strata in a 1:1 ratio for the intervention and control arms. Following the Cochrane guidelines and to reduce the possibility of recruitment bias [19], randomisation was performed by the study statistician (CL) after recruitment of clusters and before the intervention started, using a random number generator in Excel.

### Allocation concealment

Blinding of patients and programme implementers is not possible because participants know whether they are receiving a parenting or hygiene programme. However, the trial statistician carrying out randomisation and analyses is blinded, and all efforts are made to keep data collection research staff blinded as to allocation for as long as possible. Due to the nature of the intervention (i.e. families displaying home practice sheets or certificates on their walls, and children in villages singing programme songs from either Sinovuyo Teen or SinoSoap), blinding is not always maintainable. To alleviate this, audio computer-assisted self-interview (CASI) methods are used, and training of data collectors included consistent administration and awareness of biases.

### Measurement points and methods

Primary caregivers and adolescents complete measures independently and in private using a tablet with data collector support at pre-test and 3–8 months post-intervention. Tablet-based questionnaires were designed to be engaging, with embedded activities and pictures, and modified scale responses using colours etc., for participants with low literacy. All questionnaires and audio-CASI sections were pre-piloted with local adolescents and caregivers. Open-source software was used, and all questionnaires will be made freely available for other researchers.

Due to the high mobility of adolescents at the beginning of the new school year in January and the national government plans to scale the programme nationally in 2016, an additional brief immediate post-test data collection point was added, with a subset of outcome measures, to assess safety for this unanticipated scale-up. The 3–8-month post-test has been extended due to substantial levels of civil and election-related violence in the study areas, which have necessitated closing down the fieldwork for around 50 % of the time. A 15–20-month or 20–26-month post-test is planned but will depend on financial resources, especially given the unanticipated costs related to violent community protests.

To increase participant retention in the study, we will continue to hold community meetings, and to work with chieftains and local councillors in all sites prior to returning for data collection. Only data on reasons for non-participation will be collected from participants who choose not to continue in the study.

### Measures

All measures were translated into Xhosa and back-translated. Interviews take place in homes and community settings, using tablet-based questionnaires with audio-assisted interviews for stigmatised measures (child abuse, HIV/AIDS etc.). The research team are recruited locally but do not conduct data collection in their home areas, and are trained for a month on research ethics and in working with vulnerable children and families. All questionnaires are available at www.youngcarers.org.za.

### Primary outcomes

*Abusive parenting* (physical abuse, emotional abuse and neglect) is measured using an adapted version of the International Society for Prevention of Child Abuse and Neglect Child Abuse Screening Tool (ICAST-Child, 18 items, and ICAST-Parent, 22 items) [20, 21] for use in intervention studies. *Positive and involved parenting* (16 items), *monitoring and inconsistent discipline* (16 items) and *corporal punishment* (five items) are measured using the Alabama Parenting Questionnaire (parent and child versions) [22].

### Secondary outcomes

*Adolescent behaviour problems* uses the Child Behavior Checklist [23] rule-breaking and aggressive behaviour subscales (35 items). *Parenting stress* is measured using the Parental Stress Scale [24] (18 items). *Caregiver depression* (caregiver report only) is measured using the Centre for Epidemiologic Studies Depression Scale (20 items) [25]. *Adolescent depression* (adolescent report only) uses the short-form Child Depression Inventory (ten items) [26]. *Adolescent suicidality* is measured with the Mini International Neuropsychiatric Interview for Children and Adolescents [27]. *Social support* (for caregivers and adolescents) is measured using the Medical Outcomes Study Social Support Survey [28].

### Exploratory outcomes

*Family financial coping* uses items assessing shortages in monthly budgets for purchasing meat, electricity etc., and the level of emotional stress experienced as a result, plus items on capacity to respond to emergencies, and borrowing and savings behaviours [29–31]. *Planning for avoiding risk in the community* is measured using the adapted Parent Teen Sexual Risk Communication Scale III (four items) [32], and five items from the Parent Communication Scale [33]. *Exposure* is measured using items from the National Survey of HIV and Sexual Behaviour amongst Young South Africans [12, 13], sexual abuse items from the ICAST, and exposure to community violence using three items based on risks identified in the Victimisation/Witnessing of Community Violence subscales of the Social and Health Assessment (SAHA) [34, 35]. *Education* is measured using four adapted items of the SAHA academic motivation scale [36], with school attendance, grade repetition and school records of the South African standardised Annual National Assessment Learner Report if this is conducted as intended in 2016 [37]. *Attitudes towards gender norms in relationships* uses four items from the Gender Equitable Men scale [38].

### Process evaluation assessments

Process evaluation assessments include implementer records of home visits, programme attendance rates, as well as independent observations of participant engagement and implementer fidelity and adherence during sessions. In addition, focus groups with implementers, participants and data collectors explored these topics qualitatively. In the pre-test questionnaire, participants self-assessed their motivation to improve their relationship with their adolescent/caregiver, intentions to participate in both intervention and control arm programmes, and estimated difficulties and utility of attendance.

### Qualitative assessment

The collection of linked qualitative data aims to understand how policy, service delivery, and social and economic factors may impact the effectiveness and scalability of the intervention. This focuses on: (a) recommendations of local staff delivering the programme, (b) family experiences of the parenting programme in the wider context of their lives and (c) policy and programming-level considerations for scaling a parenting programme in South Africa. The qualitative research study is conducted in partnership with UNICEF’s Office of Research – Innocenti. Ongoing qualitative methods throughout the pilot and randomised trial stages (in English and Xhosa) include record analysis; semi-structured interviews with local and international NGOs, government partners and implementing partners; elite interviews with South African policymakers at the local, provincial and national levels; focus group and individual interviews with beneficiaries and implementers, including interactive activities and participatory visual methodologies; and programme workshop observation.

### Mediating and moderating pathways

Potential mediating pathways that have been shown to mediate change in primary outcomes in other studies of child abuse prevention, will be explored. Given that no known randomised controlled trials (RCTs) have tested a parenting programme for families with adolescents in a LMIC, these mediation analyses will be tentative and based on the programme’s theory of change, thus including potential mediators of change in parenting and child abuse such as parenting stress, parent mental health and social support. However, based on more substantial literature on parenting as a mediator of change in youth outcome, if there are main effects on the secondary outcomes of adolescent behavioural problems and mental health, we plan to examine changes in positive and harsh parenting as mediators of change in adolescent outcomes.

Potential moderating factors of programme effects will also be examined. Again, given the novel nature of this study, these are tentatively hypothesised. Potential moderators (see below) will include *family AIDS-illness/death*, measured using verbal autopsy/illness questionnaires validated for high-prevalence areas [39]; *poverty* measured using the National Food Consumption Survey [40] and the South African Social Attitudes Survey [41]; and *caregiver experience of maltreatment as a child* and *current gender-based violence* measured using the ICAST-Retrospective (15 items) [42], five items from the revised Conflict Tactics Scale [43] and five items from the 15-item sexual relationship power scale [44]. Potential moderators will also include *access to social protection provisions* such as grants, community gardens and soup kitchens. We prefer not to predict whether poverty, family psychosocial or illness-related factors will increase or reduce programme effects, since the parenting intervention literature is very mixed on the direction of these moderator effects [45, 46]. We will also examine variations in programme effects based on attendance and participation in the intervention, and implementer fidelity.

Potential mediation and moderation effects will be tested using structural models (either using Hayes’ PROCESS path models or structural equation modelling depending on variable type [47]), and ideally using three data points at baseline, 3–8 months post-intervention and 15–20 and 20–26 months post-intervention. If the final follow-up is not possible due to financial or practical constraints, the models will use change scores of the mediating/moderating variable from pre- to post-intervention and then analyses should clearly be interpreted with caution.

### Socio-demographic covariates

Socio-demographic covariates include age, gender, disability level, urban/rural location, household structure, caregiver–child relationship, household employment and HIV/AIDS-related stigma.

### Statistical analysis plan

The sample size calculation was conducted by YS using the ICAST child abuse measure as the basis as it is the main outcome of policy interest. We estimated the range of intra-cluster correlation coefficients up to 0.08 based on pilot testing in the study area. We note that there are a large number of zero values in the reporting of child abuse in any pragmatic sample. Using Optimal Design software [48], 40 clusters with 12 families per cluster were required for a minimum detectable effect size of 0.36 for a desired power of .80 with a significance level of 5 % with a two-tailed test. To account for attrition of up to 20 % within each cluster, the target sample size was set at 600 families (40 clusters with 15 families per cluster). Our estimate of expected effect size was based on the effect sizes for maltreatment and parenting outcomes in a recent review of parenting programmes for child maltreatment prevention [49]. This showed an average programme effect of 0.2, but the present study was limited by financial constraints, so will need to reach an effect of 0.36 to find significance.

### Types of analysis

A baseline table reflecting the demographic profile of the households, caregivers and adolescents by arm will be compiled. Statistical analyses will be by intention to treat, with an additional per-protocol analysis. For the analysis of the primary outcomes, the suitability of using linear regression models will be checked. For primary outcomes that can be analysed with this approach, a hierarchical linear mixed effects model will be used to evaluate the intervention effect with the cluster and participant (caregiver or adolescent) as the nested random effects. To account for dropouts in the intention-to-treat analysis, the baseline measurement will be part of the repeated outcomes and estimation of the intervention effects will be via maximum likelihood. The significance of the intervention effect will be based on the significance of the arm by the time interaction effect. The models will include the stratification as a fixed effect. The impact of the missing data on the estimated intervention effect will be checked by imputing missing outcome data (ten imputations) using complete baseline and follow-up data and running the same models. For a primary outcome that does not meet the criteria for an individual-level analysis, an analysis at the cluster level will be used using a standard linear regression model or a non-parametric quantile regression model. These models will allow for the comparison of arms, adjusted for the stratification and baseline cluster value. A similar sensitivity analysis will be done for the cluster-level analysis based on the imputed data. The primary and secondary outcomes will be analysed separately for caregivers and adolescents and a joint outcome model will be done as an exploratory analysis to understand the dependence of outcomes within a dyad. The impact of adherence to the intervention (number of sessions attended) will be assessed using the same models but restricted to the intervention arm.

### Stopping procedures

An independent trial steering committee has been established. Two pilot studies showed no evidence of harm [13]. If there are any indications of negative effects, as noted in process observations or participant reports, the principal investigator and partner NGOs will be alerted, and preventative action taken.

### Modifications to the protocol

Any changes to the protocol will be communicated to the relevant partners and funding bodies via email. The current protocol at the Pan-African Clinical Trials Registry will be updated online.

### Coordinating centre

The coordinating centre at the University of Oxford consists of research and academic staff. It provides administrative and scientific support and runs the fieldwork component of the research. Oxford and the South African site jointly run safety responses to political violence, through WhatsApp messaging for instant communication of unsafe areas and evacuation of staff when needed.

### Steering committee

The steering committee is composed of an independent, international group of academics with experience in running randomised trials of parenting interventions in LMICs, and child protection and child abuse prevention specialists. It provides scientific support and data monitoring where needed. The conduct of the trial is reviewed with this committee every 6 months.

### Data management

Only a small number of the research team have access to personal identifiers, which are used to match caregiver and child interviews. Using a password-protected internet network, tablet devices transmit participant responses to a password-protected server where the data are automatically captured. Questionnaires sent to the server cannot be altered. Data collected on tablets cannot be accessed unless a key is entered, which is required when they are uploaded to a central network server, which is hosted by the University of Oxford. Thus, the data are protected from both local-server failure and confidentiality breaches. Non-electronic data are stored in a locked filing cabinet. In reporting the findings of this study, names will be omitted and only general locations in which the study took place will be reported. Data will be made available at Data Archive UK or a similar open-access archive. Non-anonymised data will be kept for up to 5 years in locked cabinets.

### Capacity-building and resource-sharing

The trial aims to build capacity in low-resource contexts. This includes recruitment and intensive training of unqualified local staff, with provision of financial support for educational needs and recruitment and training of local community members in programme implementation. All study materials (qualitative and quantitative) will be freely available via the UNICEF and WHO websites. Doctoral and pre-doctoral students from LMICs are included in the study team.

### Dissemination

#### Community-level

The findings will be disseminated to all local communities, local leaders, NGOs and government departments through presentations, and a core research team will remain in the field site for 6 months to do this. At the policy and academic levels, ongoing dissemination is being carried out at academic and professional conferences and to international NGOs, LMIC governments and policymakers with regards to the development and testing of the intervention.

## Discussion

This study has potentially substantive scientific, policy and programming value. It is the first known randomised trial of a programme to prevent abuse of adolescents in a LMIC. It uses a pragmatic cluster RCT design, to provide maximum relevance to programming in low-resource settings. It is located in an area and with a population experiencing multiple concurrent challenges – and as a result of this, a number of key practical issues have emerged in beginning this trial. The study site has experienced increasing civil unrest, including violent riots related to service access, political rallies, xenophobic violence and protests against corruption. These have caused staff safety concerns, and disruptions to both data collection and intervention programming. Extended periods without electricity, drought and flooding have caused operational delays. Violent crime (hijacking of project vehicles etc.) has been rare but has required restructuring of research processes to increase staff security. Violent armed conflict between private taxi operators and petrol-bombing of roads have restricted transport of staff to field sites. Despite these challenges, the value of a pragmatic trial design is clear: increased external validity to assist decisions by policymakers. The strong interest shown by multiple countries and agencies in this programme highlights the perceived need for effective interventions to combat abuse amongst adolescents, and the importance of rigorous research evidence in this area.

## Trial status

At the time of the revised submission of this manuscript in June 2016, the trial is ongoing. Recruitment commenced in March 2015 and a total of 1108 participants (554 caregiver–adolescent dyads) have been recruited and completed baseline assessments. Despite some delays due to violent protests in programme areas, the intervention took place with all 14 sessions conducted in 19 village/peri-urban clusters and 13 sessions conducted in one cluster. Altogether, 87 % of caregivers and 85 % of adolescents received >90 % of the sessions through groups or home visits. A brief immediate post-test data collection with a subset of primary outcomes was completed in December 2015, showing a 91 % caregiver and 92 % adolescent retention rate. These immediate results were primarily to determine safety and initial effectiveness for policymakers planning the South African national scale-up. The 3- to 8-month follow-up data collection was due to complete in May 2016, but is currently delayed due to severe civil and political violence related to the 2016 local elections, and is now hoped to complete in August. The research team are currently estimating whether a 15–20-month follow-up or a 20–26-month follow-up is financially and practically possible, and if so, this would be expected to complete by December 2017. Since the submission of this manuscript, 16 countries in Southern, Eastern, Central and Northern Africa, South-East Asia and the Middle East have requested access to the programme in order to adapt it and take it to scale in their contexts (Additional file 1).

## Abbreviations

CASI, computer-assisted self-interview; ICAST, ISPCAN Child Abuse Screening Tools; ISPCAN, International Society for the Prevention of Child Abuse and Neglect; LMIC, low- and middle-income country; NGO, non-governmental organisation; PEPFAR, President’s Emergency Plan for AIDS Relief; PLH, Parenting for Lifelong Health; RCT, randomised controlled trial; SAHA, Social and Health Assessment; UNICEF, United Nations Children’s Emergency Fund; USAID, US Agency for International Development; WHO, World Health Organization

## Additional file

Additional file 1: SPIRIT 2013 checklist: recommended items to address in a clinical trial protocol. (DOC 122 KB)
